# Why do parents sign their children up for soccer in the United States?

**DOI:** 10.5114/biolsport.2026.154144

**Published:** 2025-10-01

**Authors:** José M. Oliva-Lozano, John Sullivan, Rick Cost, Felipe Lobelo, George Chiampas

**Affiliations:** 1 United States Soccer Federation, Atlanta, GA, United States; 2 Northwestern Medicine Feinberg School of Medicine, Chicago, IL, United States; 3 Hubert Department of Global Health, Rollins School of Public Health, Emory University, Atlanta, GA, United States

**Keywords:** Team Sports, Football, Youth, Child Development

## Abstract

Participation in youth sports provides children with a number of physical and psychosocial benefits. With soccer continuing to grow in the United States, understanding why families choose this sport over others can help organizations better serve their communities and promote participation. This study aimed to identify the factors influencing parents’ decisions to enroll their children in soccer in the United States. An observational, cross-sectional design was utilized, with data collected through an online survey completed by 5,052 parents and legal guardians of children aged 4 to 18. The survey included questions related to sample characteristics, extrinsic (parent-focused) benefits, child growth and development, well-being, health-related, and other factors asked in ranking and open-ended questions. Quantitative data were analyzed using descriptive statistics, while qualitative data from open-ended questions were thematically coded. Parents highly value child growth and development benefits such as discipline, respect, responsibility and handling pressure. Soccer is seen by parents as a way to keep children busy, allow for a structured schedule and to avoid trouble while using up their energy. Parents have mixed feelings about high aspirations like becoming a professional player or famous athlete, but many hope for college scholarships and personal development. Health benefits are a major reason for parents choosing to enroll their children in soccer, with a focus on maintaining a healthy lifestyle and preventing chronic illnesses. Soccer was perceived as the team sport with the greatest health benefits, showing a higher collective perception of health value compared to other sports. This study provides practical insights for soccer organizations to design programs that align with parental priorities, such as child development, health, and social interaction. It supports targeted marketing, health promotion, and educational workshops. Additionally, it helps coaches, policymakers, and community centers tailor strategies that emphasize soccer’s developmental, physical, and social benefits.

## INTRODUCTION

Participation in youth sports provides children with a number of physical and psychosocial benefits [1]. Parents often consider factors such as health promotion, enjoyment, socialization, teamwork, and personal growth when enrolling their children in sport [1]. In some cases, sport is also viewed as an outlet to keep children engaged and away from negative influences [1].

For years, researchers have been trying to understand motivational considerations for participation in youth sports. For instance, a previous study developed a parental motivation scale to examine the reasons why parents enroll their children in recreational sports [1]. Research studies have focused on a variety of factors including coaching behaviors, participation motives and sources of enjoyment [2, 3]. A majority of children initially become attracted to a sport because of influence from their friends, and most commonly remain involved in sports because of perceived enjoyment while participating in the activity [4]. This observation is supported by both sociological analyses of youth sport as a cultural phenomenon [4], and qualitative research exploring parental beliefs and motivations [3]. For example, a recent interview-based study using a social constructionist framework revealed that parents’ decisions to enroll young children in sport are shaped not only by perceived benefits, but also by broader sociocultural, political, and historical forces that frame early sport involvement as both necessary and normative [3]. Children can push their parents to allow them to be involved in sports, but often the direction comes from the parents. Parents take on a significant share of responsibility after enrolling their children in sports, including covering costs, providing transportation, and dedicating time to practices and events. Also, parents are the primary role models for their children and carry influence on their children’s motivation, behavior and psychosocial growth [5, 6].

In the United States (U.S.), there are numerous different sports for parents and children to choose from. The specific reasons why contemporary parents encourage their children to play sports and specifically choose one over the others are still not well understood. Soccer, globally referred to as football, is widely regarded as the world’s most popular sport. In the U.S., soccer has experienced increased popularity in recent decades, currently boasting an estimated 14.1 million participants according to national participation data from the National Sporting Goods Association [7]. These participation data highlight that youth involvement is especially strong: ~3.9 million participants are aged 7–11 (boys: 64.1%; girls: 35.9%) and ~3 million are aged 12–17 (boys: 53.3%; girls: 46.7%) [7]. In this regard, a book examining the geopolitical, economic, and organizational dimensions of the FIFA World Cup highlights how hosting the 1994 tournament in the United States contributed to heightened interest in the sport by positioning it within a broader framework of global visibility, media engagement, and institutional support, as the country welcomed this prestigious international sporting event [8]. Thus, hosting the next 2026 FIFA World Cup, the U.S. is poised to become the major global epicenter of competitive soccer over the next five years.

There are several potential reasons why parents may enroll their children in soccer. Over the last few decades, youth soccer has increased in popularity for a few key reasons including, (1) the relative simplicity of the rules; (2) the relative lack of expense of the equipment and uniforms; (3) the relative ease of accessibility to play for both boys and girls; and (4) finally the widely held belief that soccer is a “safer” game for children and adolescents, particularly when compared with other organized “contact sports” including football, ice hockey, and basketball [9]. In addition, playing soccer is not only positively associated with physical health adaptations, but also cognitive, motivational, and psychosocial effects [10]. These multidimensional benefits reflect the priorities parents consider when enrolling their children in recreational sports. In fact, the top three reasons reported by parents in a previous study were to develop teamwork and leadership skills (34.48%), to promote health and exercise (27.60%), and because the child wants to play (22.99%) [1].

While there is research on general motivations for youth participation in recreational sports [1, 3], including benefits related to health, enjoyment, socialization, and personal development, few studies have isolated the specific context of youth soccer and the parental motivations that shape participation in this sport. Discussions about youth sport participation often take a general perspective or emphasize the motivations of the children themselves, while less attention is typically placed on the role of parents as the primary decision-makers in early sport involvement. Understanding these motivations is essential to inform sport programming, improve communication strategies with families, and support broader efforts to leverage soccer as a tool for youth development and public health [11]. Although broader societal efforts aim to leverage sport for public health promotion, there is limited evidence on how parents perceive soccer as a vehicle for health and social development. This gap is especially relevant considering the sport’s increasing cultural visibility and participation rates in the United States, which may influence parental perceptions and decisions.

Therefore, the primary aim of this study was to identify the factors influencing parents’ decisions to enroll their children in youth soccer in the United States. These insights are intended to inform practical applications in areas such as program design, health promotion strategies, and parent engagement efforts that align with the motivations identified. Considering the findings from previous research [1, 3], the authors´ hypothesis was that parents would prioritize child development, well-being, and health-related benefits over extrinsic or parent-focused motivations when deciding to enroll their children in soccer.

## MATERIALS AND METHODS

### Study design

This is an observational and cross-sectional investigation aimed at understanding the motivations of parents for enrolling their children in soccer. An online survey was employed as the primary data collection method and responses were gathered anonymously using a secure online form. The Institutional Review Board from Northwestern University determined that this study was exempt from further review (ID: STU00220653). This study was conducted ethically according to the Declaration of Helsinki.

### Participants

A total of 5,052 parents and legal guardians voluntarily participated in the study. Informed consent was obtained from all participants prior to their inclusion in the research. The inclusion criteria for participants were as follows: 1) Parents or legal guardians of children currently enrolled in youth soccer affiliated with the U.S. Soccer Federation; 2) Children aged between 4 and 18 years, participating in leagues ranging from U5 to U18; 3) Parents or guardians aged between 21 and 70 years; 4) Residing in the United States; 5) Able to read in English. All information obtained from participants was recorded by the investigators in a manner that ensured the anonymity of the subjects, with data being de-identified.

### Data collection

An online survey was selected as the primary data collection method to allow broad, efficient, and standardized access to parents of children enrolled in youth soccer under the U.S. Soccer Federation, leveraging the contact information available through the Member Relations Department of the U.S. Soccer Federation. This approach was particularly suitable given the geographically dispersed target population and the need for a confidential and scalable data collection process. Data were collected over an 8-week period between April 2024 and May 2024. The survey was accessed through a URL link included in an email that directed participants to a Microsoft Form. Upon completion of the survey, the de-identified data were compiled into a Microsoft Excel spreadsheet, accessible only to the research team. The survey responses were categorized and stored separately for each participant.

The survey used in this study (Appendix 1) was adapted from a previous instrument conducted by Pracht et al. [1], which was originally designed to explore parents’ motivations for enrolling their children in recreational sports. In their study, Cronbach’s alpha values on the subscales were above 0.76, indicating subscale reliability with a 0.91 alpha across the items, showing strong consistency inter-item correlations. The survey was adapted by a panel of experts, including sport scientists, public health researchers, and medical doctors, to ensure content validity was maintained. These adaptations aimed to ensure contextual relevance (e.g., data on sample characteristics, health-related motivations, and other influencing factors through ranking and open-ended questions).

The survey consisted of questions related to six main content groups: (1) sample characteristics (e.g., respondent role, location, or soccer background); (2) extrinsic/parent-focused benefits (e.g., aspirations for scholarships, future opportunities, or time management); (3) child growth / development benefits (e.g., discipline, goal-setting, responsibility); (4) well-being benefits (e.g., using up extra energy or avoiding negative influences); (5) health-related benefits (e.g., maintaining a healthy lifestyle, preventing chronic illness); and (6) other factors captured through ranking (e.g., perceived health benefits compared to different sports) and open-ended questions exploring motivations and barriers. No additional tests were conducted on the adapted survey due to the reliability of the source instrument, the minimal changes made, and the large-scale nature of the data collection.

### Data Analysis

The data analysis consisted of both quantitative and qualitative approaches. Descriptive statistics, including counts, frequencies, and percentages, were used to summarize responses to closed-ended questions. The quantitative data were analyzed using SPSS (IBM, SPSS Statistics, V 29.0.1, Chicago, IL, USA). However, to analyze responses to the ranking question in which participants ordered a list of sports from most to least beneficial, each response was processed as an ordinal sequence, assigning numerical values to each position (e.g., first place = 1, second = 2, etc.). Using Python 3, the data were transformed into a long format and the mean rank was calculated for each sport. This allowed us to generate an overall ranking based on perceived health benefits. Lower average ranks indicated a higher collective perception of health benefits associated with that sport.

Finally, for open-ended questions, a qualitative analysis was performed. Open-ended responses were analyzed thematically using a hybrid approach. Initial coding and clustering were assisted by ChatGPT (OpenAI LLC, Delaware, USA), which was used to detect common terms and classify emerging themes. To ensure accuracy and preserve confidentiality, all responses were anonymous, so no personally identifiable information was processed through the AI model. Final themes were reviewed and validated manually by members of the research team to ensure interpretive consistency.

## RESULTS

The following results are presented in alignment with the main research aim: to identify the key factors that influenced parents’ decisions to enroll their children in youth soccer in the United States. Specifically, the findings are organized across core domains derived from the questionnaire structure: sample characteristics, extrinsic/ parent-focused motivations, child development benefits, well-being motivations, health-related motivations, and additional factors identified through ranking and open-ended responses that may lead to or hold back from signing up for soccer.

### Sample characteristics

Table 1 shows the characteristics of the sample participating in this study. Most respondents were mothers (62.2%), followed by fathers (36.7%). The vast majority were between 30 and 59 years old (94.2%). Children ranged from U5 to U18, with the largest representation in the U10–U12 age groups. Geographically, the most represented states were Florida (28.8%), Texas (20.8%), and Pennsylvania (13.0%). Nearly half of the parents (46.8%) had played soccer growing up, and 87.6% reported understanding the rules of the game. Similarly, 46.8% had another family member involved in soccer.

**TABLE 1:** Sample characteristics

Question	Answer	Results
Age of the parent/legal guardian	< 30 years old	1.7%
	30–59 years old	94.2%
	≥ 60 years old	1.7%
	Unknown	2.4%

Who is completing the survey?	Mother	62.2%
	Father	36.7%
	Legal guardian	1.1%

Did you grow up playing soccer?	Yes	46.8%
	No	53.2%

Do you understand the rules of soccer?	Yes	87.6%
	No	12.4%

Does anyone else in your family play soccer?	Yes	46.8%
	No	53.2%

Age group of your child/ children	U5	4.9%
	U6	5.3%
	U7	5.2%
	U8	8.9%
	U9	7.9%
	U10	12.3%
	U11	9.3%
	U12	11.5%
	U13	8.4%
	U14	8.00%
	U15	6.0%
	U16	4.9%
	U17	3.8%
	U18	3.6%

Location	Florida	28.8%
	Texas	20.8%
	Pennsylvania	13.0%
	California	8.1%
	North Carolina	7.5%
	Nebraska	5.2%
	Connecticut	4.1%
	Missouri	2.0%
	Tennessee	1.4%
	Colorado	1.2%
	Georgia	1.04%
	Rest of locations	< 1%

Regarding their personal experience with soccer, 46.8% of the respondents reported that they grew up playing soccer. In terms of understanding the rules of soccer, a significant majority (87.6%) indicated that they understand the rules, whereas 12.4% do not. When asked if anyone else in their family played soccer, 46.8% responded affirmatively.

### How important are extrinsic / parent-focused motivations?

**FIG. 1:**
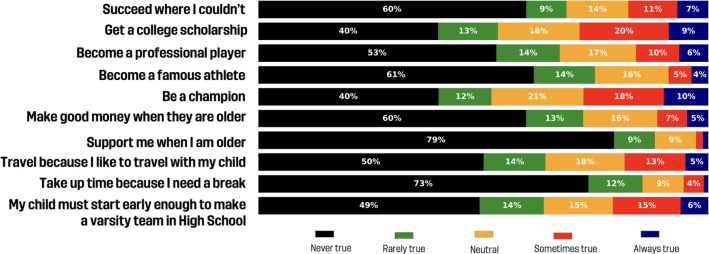
Extrinsic / Parent-focused benefits.

Figure 1 shows the results in extrinsic / parent-focused motivations. A majority of parents disagreed with statements such as “succeed where I couldn’t” (68.6% never/rarely true), support parents in older age (80.6%), or the idea of using soccer to take up time for a parental break (85.4%). Aspirations related to fame (74.9% never/rarely true), financial goals (72.2%), and professional careers (67%) were also generally dismissed. However, some parents viewed soccer as a pathway to future opportunities: 28.6% agreed it could lead to a college scholarship, and 21.2% saw early participation as a way to make a high school varsity team, and 16% viewed it as an opportunity to become professional players. The goal of their child becoming a champion showed a more mixed response: 27.7% found it always or sometimes true, while 51.8% marked it as never or rarely true. Travel motivations were also low; only 5.1% of parents reported that they always liked traveling with their child for soccer, while 63.9% said this was never or rarely true.

### How important are child growth / development motivations?

**FIG. 2:**
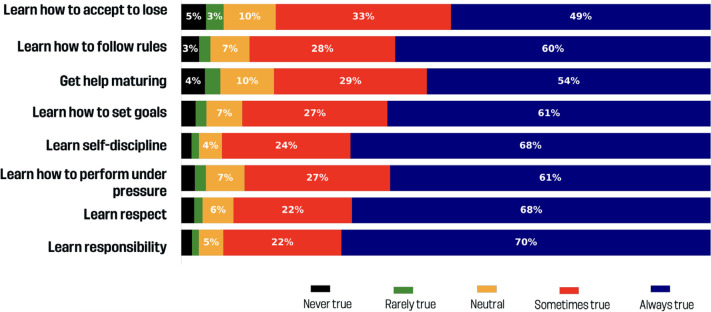
Child growth / development benefits.

Figure 2 shows the results in child growth / development motivations. Most responses to child growth / development motivations were always/sometimes true: learn how to accept to lose (82.1%), learn how to follow rules (87.1%), get help maturing (82.5%), learn how to set goals (88.5%), learn self-discipline (82.3%), learn how to perform under pressure (88.5%), learn respect (90.2%), and learn responsibility (92.1%).

### How important are well-being motivations?

**FIG. 3:**
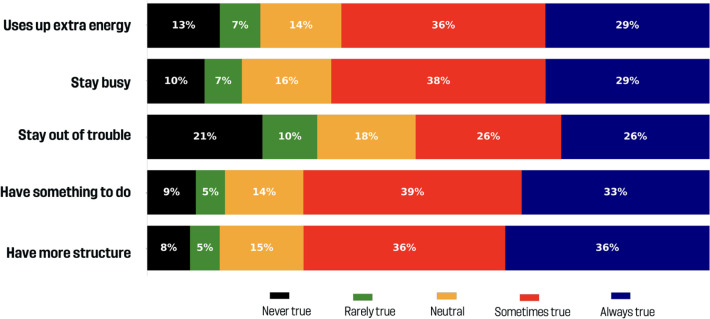
Well-being benefits.

Figure 3 shows the responses to well-being motivations. Most agreed that soccer helps children stay busy (67.3% always/sometimes true) and use up extra energy (65.5%). Similarly, 71.8% believed soccer gives children something to do, and 72.2% valued the structure it provides. The idea of preventing trouble had more mixed responses: 52.3% agreed it was a motivation, while 30.2% disagreed and 17.5% were neutral.

### How important are health-related motivations?

**FIG. 4:**
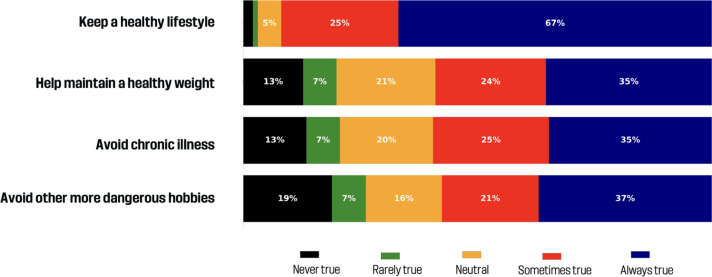
Health-related benefits.

Figure 4 shows the responses to health-related motivations. It shows that health was a key motivation for many parents. The majority (91.9%) agreed that maintaining a healthy lifestyle was a reason for enrolling their child in soccer, with 66.9% marking it as always true. Other health-related reasons were also commonly endorsed: 56.5% said helping maintain a healthy weight was always or sometimes true; 59.5% agreed with preventing chronic illness; and 57.6% considered avoiding dangerous hobbies a relevant motivation.

### Ranking of sports by most health benefits

When participants were asked to rank sports based on perceived health benefits, soccer emerged as the clear number one, with the lowest average score (1.31). It was followed by basketball (2.88), lacrosse (4.25), hockey (4.86), baseball (5.55), handball (5.61), rugby (5.63), and American football (5.92).

### Thematic analysis of factors that led to sign up for soccer

Learning and development: Parents valued soccer as a way for children to gain life skills like discipline, leadership, and resilience. For example, one respondent mentioned that they wanted their child to “learn to be a leader” and “learn to deal with adversity” while other parents made statements like “Seeing her improve her soccer skills” or “Watching him achieve his personal goals”.

Teamwork and social interaction: many parents highlighted the social benefits of soccer, including making friends and learning to work as part of a team (e.g., words like “team” “friends” and “part” were frequently observed). A typical response noted that soccer helped their child “be part of a team” suggesting that the social environment provided by the sport is a critical factor in its appeal to parents. The notion of belonging and the camaraderie fostered by team sports like soccer was a recurring theme in many of the responses.

Physical activity and health: physical activity was a common theme since soccer was seen as a fun way to stay active (e.g., keywords such as “play” and “fun” were found and/or combined in multiple responses). For example, one parent simply stated that soccer was “all about good health” while another highlighted that it was a “fun way to keep active.” This theme reflects the dual role of soccer in promoting both health and enjoyment. Parents likely view soccer as a means to keep their children physically fit and healthy, which aligns with broader societal concerns about childhood obesity and sedentary lifestyles.

Desire and motivation of the children: children’s personal interest and enthusiasm for soccer are critical drivers of participation. Parents often mentioned that their child “loved” the sport or “wanted to play,” showing that intrinsic motivation played a key role. Responses included words like “wanted” and “love” suggesting that children’s desire to play soccer played a significant role in their parents’ decision to sign them up. One parent noted, “They love it” capturing the intrinsic motivation that many children feel towards the sport.

### Thematic analysis of factors that held back from sign up for soccer

Time commitment: parents expressed concerns over busy schedules, work commitments, and the general availability required to support their child’s participation. “Time constraints” and “busy work schedules” were significant factors that prevented their child’s involvement in soccer.

Cost: the financial burden associated with soccer was a major deterrent. High costs of participation, including registration fees, equipment, and travel expenses, were key reasons for not signing up. One parent stated, “It’s too expensive,” reflecting a common sentiment that soccer may be unaffordable for many families.

Child’s interest and fit: parents indicated that their child was either not interested in soccer or did not enjoy playing. Phrases like “my child isn’t interested” or “they don’t find it fun” appear in this theme. This included concerns about social dynamics, such as “fitting in with the team” or “cultural differences” that might make soccer less appealing or accessible.

Parental attitude / influence: parental influence played a role since some parents noted that “overly competitive parents” or “loud and obnoxious parents on the sidelines” as well as their “lack of interest” or “family obligations” influenced the decision not to enroll their child in soccer.

Coaching and club quality: concerns about the quality of coaching and the overall club environment were notable due to responses such as “poor coaching” “lack of proper training” and dissatisfaction with the clubs’ organizational quality.

Competition and pressure: some parents felt that the sport was “too intense” or “focused too much on winning” which deterred them from enrolling their child.

Health and safety: parents expressed concerns related to the safety of the sport, such as the risk of injury, with comments like “worried about injuries” or “concerned about safety”.

Travel and location: the necessity to travel long distances for games or practice sessions was a barrier for some parents. Issues like “long travel distances” and “inconvenient locations” indicate that logistical challenges can influence participation.

## DISCUSSION

The findings in our study with respect to parental motivations for enrolling their children in soccer support the previous literature exploring motivations for playing sports in general [1]. Our data shows a variety of highly regarded motivating factors for parents in choosing to have their children play soccer. Parents highly value child growth and development benefits such as discipline, respect, responsibility and handling pressure. Soccer was seen by parents as a way to keep children busy, allow for a structured schedule and to avoid trouble while using up their energy. Parents have mixed feelings about high aspirations like becoming a professional player or famous athlete, but many hope for college scholarships and personal development. Health benefits were cited by parents as a major reason for choosing to enroll their children in soccer, with a focus on maintaining a healthy lifestyle and preventing chronic illnesses. Overall, soccer was believed to have the most health benefits among other team sports according to our data.

Parents placed strong value on the developmental benefits of soccer. Skills like responsibility and self-discipline were particularly emphasized, with the majority of respondents recognizing these as key outcomes of participation. The concept of self-discipline is an important one when considering the long-term implications of physical activity. One longitudinal study suggests adults that were involved in sports as a child were six times more likely to be active as adults [12]. Additionally, frequent participation in activities as a child (such as youth sports involving multiple practices or games in a week) was associated with persistent participation in activity into adulthood [12]. There are many theories to explain this, however we know that generally individuals involved in team sport are well motivated, and with this carries a sense of self-discipline and responsibility that continues to develop the more an athlete is involved in sport [12]. Therefore, it would make sense that parents would want their children to explore youth sports as a means of developing self-discipline while they are young with the hopes of carrying this into adulthood. This is also supported in the open-ended responses by parents who expressed reasons for signing up their children for soccer including “facing adversity” and “seeing potential in my child”.

Our survey demonstrated that parents likely view soccer as a means to keep their children physically fit and healthy, which aligns with broader societal concerns about childhood obesity and sedentary lifestyles. Parents rated public health benefits very highly with a majority of parents choosing always or sometimes true for all of the public health benefits, the highest rated categories being: keeping a healthy lifestyle and avoiding chronic illness. This finding is significant, as it reflects a general awareness of the value of physical activity. In line with this, previous research concluded that participating in youth sports helps children be more active and burn more energy each day, which can lead to a more active lifestyle [13–17]. Research has also shown that participation in youth sports is associated with increased physical activity levels into adulthood, suggesting participation in youth sports may lay the foundation for healthy behaviors and obesity prevention [18, 19].

Soccer is considered as a physical activity since it involves voluntary bodily movements resulting in energy expenditure and it is a form of exercise when this activity is planned, structured, and the objective is to improve or maintain physical fitness components [20]. In addition, soccer is considered as a sport because as a form of physical activity and exercise, it requires specific skills and participants may play in a competitive nature since it is organized and governed by rules [21]. A recent systematic review analyzed changes in physical fitness and health-related markers in untrained children and youths exposed to recreational soccer [22]. The main findings of the study were that supervised recreational soccer programs spanning a period of eight to eleven weeks significantly improved cardiorespiratory fitness, blood pressure, or heart rate-related variables, and it appears that these programs can be advantageous in enhancing body composition [18, 22–26]. On a global scale, through the FIFA 11 for Health program, recreational soccer has proven to be an effective way to offer an engaging activity for children as a means to improve their physical activity and understanding of prevention strategies to avoid chronic diseases in Sub-Saharan Africa [27]. Similar studies have shown this program to be efficacious in Brazil [28], China [29], Mexico [30] and other countries throughout the world. It is important for parents to recognize these potential health benefits of playing soccer as parents play an important role in encouraging their children to be and stay physically active [31].

Moreover, playing soccer is not only positively associated with fitness and health adaptations, but also cognitive, motivational, and psychosocial effects. This can be seen through various aspects of sport such as discipline, working as a team, being a leader, facing adversity, adhering to a schedule, helping to mature and avoiding dangerous hobbies which is demonstrated in our data. In our data, more than 75% of parents chose always or sometimes true for the categories involving child growth and development. Studies have shown that exercise has positive impacts on a person’s wellbeing, improving cognitive function and reducing anxiety [32]. A recent systematic review demonstrated that athletes involved in youth sports reported higher social functioning, mental health and happiness when compared to non-athletes [33]. Our data suggests that parents highly regard not only the physical benefits but also the psychosocial benefits associated with playing soccer.

In response to the open-ended questions, parents commonly highlighted their child’s enjoyment, desire to play, and the opportunity for physical activity, socialization, and personal development as key reasons for choosing soccer. A previous study observed that parents’ primary motivators for enrolling their children in recreational sports include the child’s gain in life skills, well-being, and the child’s own intrinsic motivation to participate [1]. Parents seem to value the child’s ability to enjoy the activity as well as the anticipated benefits the child will gain from participating in the sport [1].

Extrinsic or parent-focused benefits were rated the least important motivations (e.g., future support or taking a break) and were largely dismissed. This is consistent with previous literature related to parents’ motivations for enrolling children in all recreational sports which suggests parents are primarily driven by the intrinsic interests of the child instead of extrinsic factors [1]. In line with self-determination theory, parents valued factors that were intrinsic to the child (physical and psychosocial benefits) over extrinsic or parent-focused benefits. Previous research suggests that intrinsic motivation is related with positive outcomes in education and sport domains [34]. Conceptually, parents would want to choose an activity or sport that their child would enjoy and benefit from in a variety of ways. Also, if the child also perceives these benefits, self-determination theory would suggest it will lead to motivation for longevity in the sport [34].

When participants were asked to rank sports based on what they believed provided the greatest health benefits, soccer was rated first while American football was rated last. Some of the parents’ biggest concerns that held them back from signing their children up for soccer included fear of injury to the child, time or money constraints and the stress or pressure the child may experience related to playing soccer. Previous literature has suggested that the risk of injury in soccer is lower than that of other popular sports in the U.S. such as football or basketball [35]. For example, it has been showed that the highest team-sport injury rate was basketball (14.4/1000), followed by football (8.4/1000) and soccer (5.2/1000) [35].

This study has several limitations that should be considered when interpreting the findings. First, the survey participants were selected from a group of parents that currently have their children enrolled in the U.S. Soccer system leading to selection bias when considering opinions of parents that do not have children enrolled in soccer. The study population may perceive the benefits of soccer differently than those who are not exposed to soccer or who have never played the game before. Parents without children who play soccer may have different perceptions of the risks of playing. That being said, this may lead to room for others to learn about the benefits of soccer who may not be familiar with the sport. A potential future study could involve surveying parents of children enrolled in other common sports in the U.S. such as baseball, basketball or football with the same set of questions to analyze whether parents’ motivations are similar or vary across different sports. Another limitation is that our current data includes parents of all age groups and an interesting future study could include subset groups of varying ages to identify if motivations are different across various age ranges. Future analysis could be done to stratify groups of parents who played soccer compared to those who haven’t and compare answers. Moreover, while the survey instrument was based on a previously used tool [1] and its content validity was reviewed by experts to be adapted to the context of soccer, the lack of revalidation in this specific context limits the ability to assess the reliability of the adapted instrument.

Although our data lacks external validity at an international level as participants are exclusively involved in soccer within the U.S., our large study population of parents across the U.S. Soccer system offers valuable information in understanding parents’ motivations for enrolling children in soccer. This knowledge can be used to raise awareness to other adults and children about the benefits of playing soccer to ultimately promote the sport and have a positive impact on individuals physical and mental health.

This study has significant practical implications. Soccer organizations and clubs can use the findings to tailor programs that emphasize the benefits parents prioritize, such as child development, health, and social interaction. This can help attract more families to enroll their children in soccer programs. Also, health promotion campaigns through soccer may be designed as the study highlights soccer’s health and fitness benefits, making it a valuable tool for public health campaigns that aim to combat childhood obesity, sedentary lifestyles, and promote physical activity among children. Moreover, for parental engagement, soccer clubs and youth sports organizations can create targeted communications and marketing strategies addressing the concerns and motivations of parents, including providing affordable, accessible, and developmental programs that align with their expectations. This may include educational workshops for parents, focusing on how soccer helps develop life skills, maintain health, and foster positive social interactions, reinforcing the value of their involvement in the sport.

In addition, governing bodies can use the study to advocate for more resources and funding for youth soccer, positioning it as a sport that offers broad physical, social, and emotional benefits for children. Also, there may be youth coaching strategies since coaches can better understand parental motivations and focus on teaching life skills like discipline, teamwork, and resilience, aligning their training methods with the expectations of families, which may lead to higher retention rates in youth soccer. Finally, schools and community centers can collaborate with local soccer programs to provide structured activities that support both physical and social development, using soccer as a tool for broader educational and social purposes.

## CONCLUSIONS

This study highlights the multifaceted motivations of parents when enrolling their children in soccer. Key findings indicate that beyond athletic aspirations, parents highly value soccer as a means to support their children’s overall development, including discipline, responsibility, and social integration. Health-related benefits and the promotion of an active lifestyle also emerged as strong motivators. While ambitions such as professional careers were viewed with caution, educational opportunities and personal growth were broadly endorsed. These insights underscore the potential of soccer not only as a sport but also as a developmental and health-promoting tool. Future programs and policies that align with parental priorities may enhance participation, retention, and the broader impact of youth soccer on child well-being.

## Supplementary Material

Why do parents sign their children up for soccer in the United States?

## References

[1] Pracht DW, Houghton V, Fogarty K, Sagas M. Parents’ motivations for enrolling their children in recreational sports. J Amat Sport. 2020 Mar 13; 6(1):81–99.

[2] McCullagh P, Matzkanin KT, Shaw SD, Maldonado M. Motivation for participation in physical activity: a comparison of parent–child perceived competencies and participation motives. Pediatr Exerc Sci. 1993 Aug; 5(3):224–33.

[3] Mysko E, Elliott S, Drummond M. Understanding parents’ motives for, and beliefs about, enrolling three-to-five-yearold children into organised sporting programs. Qual Res Sport Exerc Health. 2023 Jul 4; 15(4):481–500.

[4] Woods RB. Social issues in sport. Human Kinetics; 2011.

[5] Holt NL, Tamminen KA, Black DE, Mandigo JL, Fox KR. Youth sport parenting styles and practices. J Sport Exerc Psychol. 2009 Feb; 31(1):37–59. 19325187 10.1123/jsep.31.1.37

[6] Dorsch TE, Wright E, Eckardt VC, Elliott S, Thrower SN, Knight CJ. A history of parent involvement in organized youth sport: A scoping review. Sport Exerc Perform Psychol. 2021 Nov; 10(4):536–57.

[7] National Sporting Goods Association. Sports participation in the United States. Downers Grove, IL; 2022.

[8] Chadwick S, Widdop P, Anagnostopoulos C, Parnell D. The Business of the FIFA World Cup. New York: Routledge; 2022.

[9] Metzl JD, Micheli LJ. Youth soccer: an epidemiological perspective. Clin Sports Med. 1998 Oct; 17(4):663–73. 9922893 10.1016/s0278-5919(05)70110-1

[10] Jonker L, Elferink-Gemser MT, Toering TT, Lyons J, Visscher C. Academic performance and self-regulatory skills in elite youth soccer players. J Sports Sci. 2010 Dec; 28(14):1605–14. 21104520 10.1080/02640414.2010.516270

[11] Oliva-Lozano JM, Chiampas GT, Cost R, Sullivan J, Lobelo F. Elevating recreational soccer to improve population health in the United States: the time is now. Front Public Health. 2024 Oct 18; 12:1–5. 10.3389/fpubh.2024.1406878 PMC11527682 39494074

[12] Telama R, Yang X, Hirvensalo M, Raitakari O. Participation in organized youth sport as a predictor of adult physical activity: a 21-year longitudinal study. Pediatr Exerc Sci. 2006 Mar; 18(1):76–88.

[13] Lee JE, Pope Z, Gao Z. The role of youth sports in promoting children’s physical activity and preventing pediatric obesity: a systematic review. Behavioral Medicine. 2018 Jan 2; 44(1):62–76. 27337530 10.1080/08964289.2016.1193462

[14] Weintraub DL, Tirumalai EC, Haydel KF, Fujimoto M, Fulton JE, Robinson TN. Team sports for overweight children. Arch Pediatr Adolesc Med. 2008 Mar 1; 162(3):232–237. 18316660 10.1001/archpediatrics.2007.43

[15] Machado-Rodrigues AM, Silva MJC, Mota J, Santos RM, Cumming SP, Malina RM. Physical activity and energy expenditure in adolescent male sport participants and nonparticipants aged 13 to 16 years. J Phys Act Health. 2012 Jul; 9(5):626–33. 21953445 10.1123/jpah.9.5.626

[16] Katzmarzyk PT, Malina RM. Contribution of organized sports participation to estimated daily energy expenditure in youth. Pediatr Exerc Sci. 1998 Nov; 10(4):378–86.

[17] Leek D, Carlson JA, Cain KL, Henrichon S, Rosenberg D, Patrick K, Sallis JF. Physical activity during youth sports practices. Arch Pediatr Adolesc Med. 2011 Apr;165(4):294–299. 21135319 10.1001/archpediatrics.2010.252

[18] Kjønniksen L, Anderssen N, Wold B. Organized youth sport as a predictor of physical activity in adulthood. Scand J Med Sci Sports. 2009 Oct 30; 19(5):646–54. 18694430 10.1111/j.1600-0838.2008.00850.x

[19] Tammelin T, Näyhä S, Hills AP, Järvelin MR. Adolescent participation in sports and adult physical activity. Am J Prev Med. 2003 Jan; 24(1):22–8. 12554020 10.1016/s0749-3797(02)00575-5

[20] Caspersen CJ, Powell KE, Christenson GM. Physical activity, exercise, and physical fitness: definitions and distinctions for health-related research. Public health reports [Internet]. 1985; 100(2):126–31. Available from: https://www.ncbi.nlm.nih.gov/pubmed/3920711. 3920711 PMC1424733

[21] Jenny SE, Manning RD, Keiper MC, Olrich TW. Virtual(ly) athletes: where eSports fit within the definition of “sport.” Quest. 2017 Jan 2; 69(1):1–18.

[22] Clemente FM, Moran J, Ramirez-Campillo R, Oliveira R, Brito J, Silva AF, Badicu G, Praça G, Sarmento H. Recreational soccer training effects on pediatric populations physical fitness and health: a systematic review. Children. 2022 Nov 18; 9(11):1–23. 10.3390/children9111776 PMC9689246 36421225

[23] Hammami A, Randers MB, Kasmi S, Razgallah M, Tabka Z, Chamari K, Bouhlel E. Effects of soccer training on health-related physical fitness measures in male adolescents. J Sport Health Sci. 2018 Apr; 7(2):169–75. 30356474 10.1016/j.jshs.2017.10.009 PMC6180556

[24] Wang J, Cao L, Xie P, Wang J. Recreational football training improved health-related physical fitness in 9- to 10-year-old boys. J Sports Med Phys Fitness. 2018 Feb; 58(3):326–31. 27792217 10.23736/S0022-4707.16.06620-2

[25] Larsen MN, Nielsen CM, Madsen M, Manniche V, Hansen L, Bangsbo J, Krustrup P, Hansen PR. Cardiovascular adaptations after 10 months of intense school-based physical training for 8- to 10-year-old children. Scand J Med Sci Sports. 2018 Aug 26; 28(S1):33–41. 30047176 10.1111/sms.13253

[26] Cvetković N, Stojanović E, Stojiljković N, Nikolić D, Scanlan AT, Milanović Z. Exercise training in overweight and obese children: Recreational football and high-intensity interval training provide similar benefits to physical fitness. Scand J Med Sci Sports. 2018 Aug 6; 28(S1):18–32. 29979479 10.1111/sms.13241

[27] Fuller CW, Junge A, DeCelles J, Donald J, Jankelowitz R, Dvorak J. ‘Football for Health’—a football-based healthpromotion programme for children in South Africa: a parallel cohort study. Br J Sports Med. 2010 Jun 14; 44(8):546–54. 20547667 10.1136/bjsm.2010.072223 PMC2938885

[28] Fuller CW, Thiele ES, Flores M, Junge A, Netto D, Dvorak J. A successful nationwide implementation of the ‘FIFA 11 for Health’ programme in Brazilian elementary schools. Br J Sports Med. 2015 May; 49(9):623–9. 25805807 10.1136/bjsports-2015-094767 PMC4413689

[29] Li Z, Krustrup P, Randers MB, Xu B, Yang W, Huang Z, Mao L. “11 for Health” in China – Effects on physical fitness in 9–11-year-old schoolchildren. Eur J Sport Sci. 2023 Dec; 23(12):2291–8. 37338107 10.1080/17461391.2023.2227139

[30] Barriguete Melendez JA, Dvorak J, Córdova Villalobos J, Juan Lopez M, Davila Torres J, Compeán Palacios J, Valdés-Olmedo J, Junge A, Fuller C. FIFA 11 for Health in Mexico: a school-based intervention for the prevention of obesity and non-communicable diseases. Br J Sports Med. 2014 Jun; 48(12):940–1. 23613515 10.1136/bjsports-2013-092449

[31] Brockman R, Jago R, Fox KR, Thompson JL, Cartwright K, Page AS. “Get off the sofa and go and play”: Family and socioeconomic influences on the physical activity of 10–11 year old children. BMC Public Health. 2009 Dec 21; 9(1):253. 19622143 10.1186/1471-2458-9-253 PMC2718886

[32] Taylor CB, Sallis JF, Needle R. The relation of physical activity and exercise to mental health. Public Health Reports. 1985; 100(2):195–202. 3920718 PMC1424736

[33] Eather N, Wade L, Pankowiak A, Eime R. The impact of sports participation on mental health and social outcomes in adults: a systematic review and the ‘Mental Health through Sport’ conceptual model. Syst Rev. 2023 Jun 21; 12(1):102. 37344901 10.1186/s13643-023-02264-8 PMC10286465

[34] Vallerand RJ. Toward A Hierarchical Model of Intrinsic and Extrinsic Motivation. In: Advances in experimental social psychology. Academic Press, 1997. p. 271–360.

[35] Carter EA, Westerman BJ, Hunting KL. Risk of injury in basketball, football, and soccer players, ages 15 years and older, 2003–2007. J Athl Train. 2011 Sep 1; 46(5):484–8. 22488135 10.4085/1062-6050-46.5.484 PMC3418954

