# Supplementary material for: Why do parents sign their children up for soccer in the United States?
Source: Biol Sport. 2025 Oct 1;43:429–38. doi: 10.5114/biolsport.2026.154144 (PMC12954489; doi:10.5114/biolsport.2026.154144)
Supplement: Why do parents sign their children up for soccer in the United States? [file JBS-43-56663-s1.pdf]

Supplementary Material

APPENDIX TABLE 1. List of questions included in the survey

Compo-nent	Question	Answer
Sample Characteristics	Is your child currently enrolled in soccer?	Yes / No
	Location	State
	Age of the parent/legal guardian	Years old
	Who is completing the survey?	Mother / Father / Legal Guardian
	Age group of your child/children	Select from U5 to U18
	Did you grow up playing soccer?	Yes / No
	Does anyone else in your family play soccer?	Yes / No
	Do you understand the rules of soccer?	Yes / No
Extrinsic / Parent-Focused Benefits	Succeed where I couldn't	**Likert scale:** Never true, Rarely true, Neutral, Sometimes true, Always true
	Get a college scholarship	
	Become a professional player	
	Become a famous athlete	
	Be a champion	
	Make good money when they are older	
	Support me when I'm older	
	Travel because I like to travel with my child	
	Take up time because I need a break	
	Because my child must start early enough to make a varsity team in High School	
Child Growth / Development Benefits	Learn how to accept to lose	**Likert scale:** Never true, Rarely true, Neutral, Sometimes true, Always true
	Learn how to follow rules	
	Learn how to mature	
	Learn how to set goals	
	Learn self-discipline	
	Learn how to perform under pressure	
	Learn respect	
	Learn responsibility	
Well-Being Benefits	Use up extra energy	**Likert scale:** Never true, Rarely true, Neutral, Sometimes true, Always true
	Stay busy	
	Stay out of trouble	
	Have something to do	
	Have more structure	
Health-Related Questions	Keep a healthy lifestyle	**Likert scale:** Never true, Rarely true, Neutral, Sometimes true, Always true
	Help maintain a healthy weight	
	Avoid chronic illness (such as obesity, heart disease, and diabetes)	
	Avoid other more dangerous hobbies (e.g., drug use, sexually transmitted diseases, etc.)	
Other Components	Please, rank the following sports by which you believe have the most health benefits.	Soccer, Basketball, Baseball, Hockey (ice, field, roller, and street hockey), Lacrosse, Handball, Rugby, American football
	List some other factors that led you to sign your child/children up for soccer:	Open-ended questions
	List some other factors that held you back from signing your child up for soccer	Open-ended questions
